# Development of pH-Responsive Polymer Coating as an Alternative to Enzyme-Based Stem Cell Dissociation for Cell Therapy

**DOI:** 10.3390/ma14030491

**Published:** 2021-01-20

**Authors:** Yu-Jin Kim, Tae-Jin Lee, Gun-Jae Jeong, Jihun Song, Taekyung Yu, Doo Sung Lee, Suk Ho Bhang

**Affiliations:** 1 School of Chemical Engineering, Sungkyunkwan University, Suwon 16419, Korea; yujinkim1003@gmail.com (Y.-J.K.); leetj@kangwon.ac.kr (T.-J.L.); jih2616@naver.com (J.S.); dslee@skku.edu (D.S.L.); 2 Department of Bio-Health Convergence, Kangwon National University, Chuncheon 24341, Korea; 3 School of Biological Sciences, Georgia Institute of Technology, Atlanta, GA 30332, USA; jgj814@gmail.com; 4 Department of Chemical Engineering, Kyung Hee University, Youngin 17104, Korea; tkyu@khu.ac.kr; 5 Theranostic Macromolecules Research Center, Sungkyunkwan University, Suwon 16419, Korea

**Keywords:** angiogenesis, enzyme free cell detachment, hindlimb ischemia, human adipose tissue-derived stem cells, pH-sensitive polymer

## Abstract

Cell therapy usually accompanies cell detachment as an essential process in cell culture and cell collection for transplantation. However, conventional methods based on enzymatic cell detachment can cause cellular damage including cell death and senescence during the routine cell detaching step due to an inappropriate handing. The aim of the current study is to apply the pH-responsive degradation property of poly (amino ester) to the surface of a cell culture dish to provide a simple and easy alternative method for cell detachment that can substitute the conventional enzyme treatment. In this study, poly (amino ester) was modified (cell detachable polymer, CDP) to show appropriate pH-responsive degradation under mild acidic conditions (0.05% (*w*/*v*) CDP, pH 6.0) to detach stem cells (human adipose tissue-derived stem cells (hADSCs)) perfectly within a short period (less than 10 min). Compared to conventional enzymatic cell detachment, hADSCs cultured on and detached from a CDP-coated cell culture dish showed similar cellular properties. We further performed in vivo experiments on a mouse hindlimb ischemia model (1.0 × 10^6^ cells per limb). The in vivo results indicated that hADSCs retrieved from normal cell culture dishes and CDP-coated cell culture dishes showed analogous therapeutic angiogenesis. In conclusion, CDP could be applied to a pH-responsive cell detachment system as a simple and easy nonenzymatic method for stem cell culture and various cell therapies.

## 1. Introduction

Stem cell detachment from cell culture dishes is an essential step in the cell culture process and cell collection for stem cell therapies [1,2]. Despite the prominent therapeutic results of stem cell therapies, conventional cell detaching methods based on enzymatic cell dissociation still contains intrinsic disadvantages such as stem cell death and senescence caused by an inappropriate handing [3,4]. Therefore, detaching stem cells from the cell culture dish without cellular damage remains a basic and prime issue to be solved. Various trials including light- [5], temperature- [6,7], and pH-responsive [8,9,10] strategies for detaching stem cells from the cell culture dish without using enzymes have been reported. However, previous reports usually require complicated steps, tailored equipment, and long operation time for stem cell detachment. Therefore, a simple and easy method that can be suggested as an alternative method for enzymatic cell dissociation is required.

**Figure 1:**
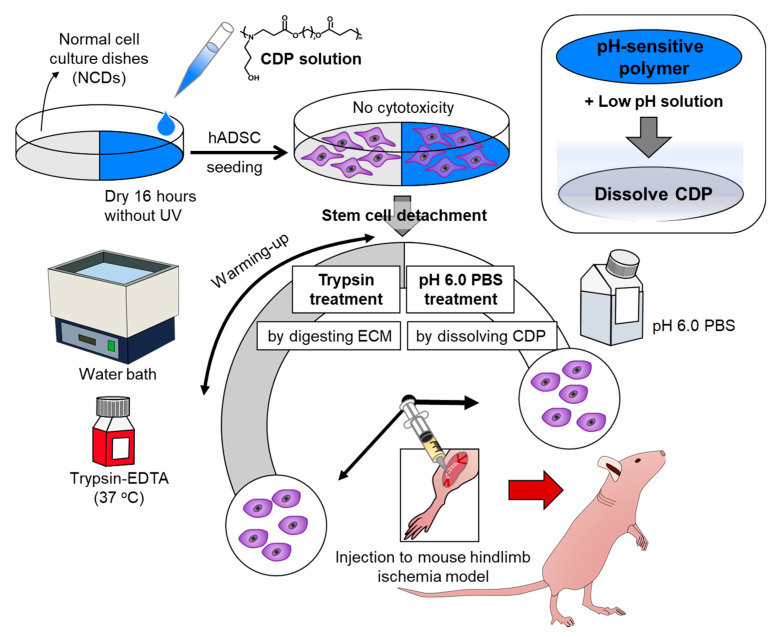
Schematic diagram depicting cell detachable polymer (CDP)-based stem cell detachment. Schematic describing the preparation of human adipose tissue-derived stem cells (hADSCs) detached from normal cell culture dishes (NCD) with trypsin treatment by digesting ECM and hADSCs cultured on and dissociated from CDP-coated culture dishes with pH 6.0 PBS treatment by dissolving CDP for ischemic disease treatment.

A pH-sensitive polymer such as poly (amino ester) (PAE) is not only simple and easy to utilize without special processes, but also has eminent biocompatibility [11]. Thus, PAE has been reported as a transplantable synthetic material that can be utilized as an enteric gene delivery system with a high gene delivery efficiency [12,13]. Additionally, PAE has been reported to show rapid degradation under an acidic environment [14]. Based on the excellent pH-sensitive degradation and biocompatibility, we modified the PAE and applied it as a nonenzymatic cell detaching polymer (CDP). In this study, we modified the PAE with Michael addition polymerization as a cell detaching material that can be coated on a normal cell culture dish (NCD) and degrade away by treating pH 6.0 phosphate-buffered saline (PBS). The segments utilized in the polymerization induced polymer degradation based on the static electric repulsion under pH 6.0 conditions and stability during the cell culture. We coated our CDP on NCDs and cultured human adipose tissue-derived stem cells (hADSCs), to investigate whether our material can show similar cellular properties compared to conventional enzymatic cell detachment systems using trypsin-ethylene diamine tetra acetic acid (EDTA). Cellular properties such as cell adhesion, viability, apoptotic activity, and angiogenic paracrine factor secretion before and after the cell detachment based on CDP showed similar results compared to the trypsin-EDTA method. Further in vivo experiments using a mouse hindlimb ischemia model also showed similar therapeutic efficacy including angiogenesis, blood perfusion, limb salvage, and hampered muscle degeneration in hADSCs retrieved from CDP-coated cell culture dishes compared to those of trypsin-EDTA treatment (Figure 1).

## 2. Materials and Methods

### 2.1. Materials

Sodium chloride, sodium phosphate dibasic, sodium phosphate monobasic, potassium chloride, 1,4-butanediol diacrylate (BD), and 3-amino-1-propanol (AP) were obtained from Sigma (St. Louis, MO, USA) and used as received. Sodium hydroxide solutions (NaOH), hydrochloric acid solutions (HCl), dichloromethane (DCM), and ethyl ether were all purchased from Samchun (Seoul, Korea). All reagents were of analytical grade and used without further purification.

### 2.2. CDP Synthesis and Characterization

The pH-sensitive cationic polymer CDP was synthesized using Michael polymerization as the method referred before [15]. Briefly, the reaction mixing BD and AP in a molar ratio of 1:1 was carried out at 100 °C for 5 h. The product, a viscous liquid, dissolved in DCM and then precipitated in excess ethyl ether. The product was dried in vacuum for 2 days, and then the molecular structure and composition of the CDP were confirmed by an ^1^H NMR spectrometer (Varian Unity Inova 500 NB instrument, Varian Co., Palo Alto, CA, USA) operated at 500 MHz. DMSO (DMSO-d_6_) was used as solvent. The conversion between monomer A, B, and CDP was confirmed by ^1^H NMR. In detail, the integrated area of each peak in NMR is proportional to two parameters, which are the number of hydrogen atoms on the functional group and repetends of the corresponding functional group. Thus, NMR analysis in the mixture of products and reactants gave information about the mass ratio of the particular components composed of the specific functional group [16]. The Mw and its polydispersity index were measured by gel permeation chromatography (GPC) using a PL aquagel-OH mixed-H column (Agilent, Santa Clara, CA, USA) and an Agilent 110 series system (Agilent, Santa Clara, CA, USA) with a refractive index detector (G1362A RID) at a flow rate of 1.0 mL/min (eluent: THF: 35 °C). Poly (ethylene glycol) was used as a standard. The pH-sensitivity (pKa) of the CDP was evaluated using acid–base titration. Briefly, 50 mg CDP was dissolved in 50 mL distilled water and the pH of the solution was adjusted to pH 3 by adding 1 N HCl to distilled water. The titration file was obtained by recording pH changes facilitated by the stepwise addition of 10 μL 0.1 N NaOH. The pKa value of CDP was calculated from the derivative of the titration curve [17].

### 2.3. Cell Culture

Human adipose tissue-derived stem cells (hADSCs) were purchased form Lonza (Walkersville, MD, USA) and cultured in Dulbecco’s modified Eagle’s medium (DMEM: Gibco BRL, Waltham, MA, USA) supplemented with 1% (*v*/*v*) penicillin (Gibco BRL, Waltham, MA, USA), and 10% (*v*/*v*) fetal bovine serum (Gibco BRL, Waltham, MA, USA). All experiments were performed on hADSCs with less than six passages.

### 2.4. CDP Coating on Cell Culture Dishes

CDP was dissolved in distilled water (pH 3.0) at 0.05% (*w*/*v*) at room temperature. After dissolving the CDP, the pH was adjusted to 7.0 with NaOH and filtered for sterilization (0.22 μm filter, Corning Inc., New York, NY, USA). To coat the cell culture dishes, the CDP solution was poured into the dishes (0.5 mL/well in 24-well, 1 mL/well in 6-well, 5 mL for 100 mm culture dishes, and 8 mL for 150 mm culture dishes) and dried on a clean bench overnight without ultraviolet irradiation. The CDP-coated culture dishes were then washed three times with PBS (Gibco BRL, Waltham, MA, USA) before their use in cell culture.

### 2.5. Cells Culture on CDP-Coated Culture Dishes and Dissociation from CDP-Coated Culture Dishes Using pH 6.0 PBS

hADSCs were seeded on CDP-coated or noncoated culture dishes. One day after seeding, the cells were washed with PBS and then incubated with 10 mL of pH 6.0 PBS (P0219, CUREBIO, Seoul, Korea) at 37 °C for 10 min. The number of detached hADSCs was counted using a hemocytometer. hADSCs detached from NCDs using trypsin-EDTA (Gibco BRL, Waltham, MA, USA) were used as a control.

### 2.6. Phalloidin Staining

Cell adhesion was examined by phalloidin staining. hADSCs were seeded on a CDP-coated or noncoated 24-well (1.5 × 10^4^ cells/well). After one day, cells were fixed with 4% paraformaldehyde (Biosesang, Sungnam, Korea) in PBS for 10 min at room temperature. Cell re-adhesion was also examined by phalloidin staining. For the re-adhesion test, 1.5 × 10^4^ cells that dissociated from CDP-coated culture dishes using pH 6.0 PBS or from NCDs using trypsin-EDTA were re-seeded in 24-well culture dishes. Cells were fixed with 4% paraformaldehyde (Biosesang, Sungnam, Korea) in PBS for 10 min at room temperature. For phalloidin staining, the fixed hADSCs were stained with TRITC-phalloidin containing mounting medium (VECTASHIELD H-1600, Vector, Burlingame, CA, USA), counter-stained with 4′,6-diamidino-2-phenylindole (DAPI, Vector, Burlingame, CA, USA), and then examined using a confocal laser scanning microscope (ZEISS, Munich, Germany).

### 2.7. Cell Growth Rate

Cell growth rate was analyzed using cell counting kit-8 (CCK-8; Dojindo Molecular Technologies, Inc., Kumamoto, Japan). For the cell growth rate assays, the hADSCs were seeded on CDP-coated or noncoated culture dishes (24-well, 5 × 10^3^ cells/well), and cell growth rate was measured using the CCK-8 kit on days 1, 3, 5, and 7. At each time point, culture medium with 10% (*v*/*v*) CCK-8 solution was added to each well, and the plates were incubated for 3 h at 37 °C. Then, the absorbance of each well was measured at 450 nm (Infinite F50, TECAN, Männedorf, Switzerland).

### 2.8. Live/Dead Assay

The cell viability of the hADSCs cultured on the CDP-coated culture dishes was analyzed using FDA/EB staining. For this assay, the hADSCs were seeded on the CDP-coated or noncoated culture dishes (24-well, 1.5 × 10^4^ cells/well), and the cells were treated with fluorescein diacetate (FDA; Sigma, St. Louis, MO, USA) and ethidium bromide (EB; Sigma, St. Louis, MO, USA) 24 h after seeding. The hADSCs were incubated in the FDA/EB solution for 5 min at 37 °C and then washed twice with PBS according to the manufacturer’s instructions. Dead cells were stained red owing to the nuclear permeability of EB, whereas viable cells, capable of converting the nonfluorescent FDA into fluorescein, were stained green. After staining, the samples were examined using a fluorescence microscope (Nikon TE2000, Nikon, Tokyo, Japan).

### 2.9. In Vitro Quantitative Real-Time Polymerase Chain Reaction (qRT PCR) Analysis

The apoptotic activity of the hADSCs cultured on the CDP-coated culture dishes and the apoptotic activity and angiogenic growth factor secretion of the hADSCs detached from CDP with pH 6.0 PBS were evaluated by qRT-PCR. For qRT-PCR analysis, the hADSCs were seeded on the CDP-coated or noncoated culture dishes (1 × 10^5^ cells/100 mm culture dishes). The expression of human *BCL2* and *BAX* mRNA was evaluated to check the anti- and pro-apoptotic activity of the hADSCs, respectively, whereas the expression of human vascular endothelial growth factor (*VEGF*) and basic fibroblast growth factor (*FGF2*) was used to detect angiogenic factor secretion in the hADSCs. qRT-PCR was performed on a CFX connect^TM^ real-time system (Bio-Rad Laboratories, Hercules, CA, USA) with a SsoAdvanced^TM^ Universal SYBR^®^ Green Supermix (Bio-Rad Laboratories, Hercules, CA, USA). The primer sequences are shown in Table 1. hADSCs cultured on NCDs or trypsin-EDTA-treated hADSCs cultured on NCDs were used as the control.

### 2.10. Treatment of Hindlimb Ischemia

Hindlimb ischemia was induced in mix as previously described [18]. Four-week-old, female athymic mice (20–25 g body weight, Orient Bio Inc., Sungnam, Korea) were anesthetized with an intraperitoneal injection of xylazine (10 mg/kg) and ketamine (100 mg/kg). The femoral artery and its branches were ligated via skin incision using a 6–0 silk suture (Ethicon, Somerville, NJ, USA), along with the external iliac artery and all upstream arteries. The femoral artery was then excised from its proximal origin as a branch of the external iliac artery to the distal point whereupon it bifurcates into the saphenous and popliteal arteries. After arterial dissection, mice were randomly divided into three experimental groups (*n* = 5 mice per group). Normal cell culture dish—cultured hADSCs (NCD, 1.0 × 10^6^ cells per limb) and CDP—coated culture dish-cultured hADSCs (CDP, 1.0 × 10^6^ cells per limb) were intramuscularly injected into the gracilis muscle of the medial thigh. The physiological status of ischemic limbs was followed for 4 weeks after treatment. The no-treatment group was used as a negative control. All animal experiments were carried out in accordance with the guidelines of the Animal Welfare Act and the Guide for the Care and Use of Laboratory Animals, following protocols approved by the Institutional Animal Care and Use Committee (Sungkyunkwan University School of Medicine SKKUIACUC2020-06-11-1). All mice used in the experiments were cared for under specific-pathogen-free conditions.

### 2.11. Laser Doppler Imaging Analysis and In Vivo qRT PCR Analysis

A laser Doppler perfusion imager (Moor Instruments, Devon, UK) was used for serial noninvasive physiological evaluation of neovascularization. Mice were monitored by serial scanning of surface blood flow in hindlimbs on days 0, 7, 14, 21, and 28 after treatment. Digital color-coded images were scanned and analyzed to quantify blood flow in ischemic regions from the knee joint to the toe. Mean values of perfusion were subsequently calculated.

For qRT-PCR, total RNA was extracted from the retrieved ischemic limb tissue (*n* = 4 per group). RNA was reverse-transcribed into cDNA. The expression of mouse platelet endothelial cell adhesion molecule (*Pecam-1*) and vascular cell adhesion molecule (*Vcam*) mRNA was evaluated by qRT-PCR. qRT-PCR was performed using a CFX connect^TM^ real-time system (Bio-Rad Laboratories, Hercules, CA, USA) with SsoAdvanced^TM^ Universal SYBR^®^ Green Supermix (Bio-Rad Laboratories, Hercules, CA, USA). Each cycle consisted of the following times and temperatures: 95 °C for 10 s and 60 °C for 30 s. The primer sequences are shown in Table 1.

### 2.12. Histology and Immunohistochemistry

Ischemic limb muscles were retrieved 28 days post-treatment. The tissues were fixed with 4% paraformaldehyde (Biosesang) in PBS and embedded in an optimal cutting temperature compound (O.C.T. compound, Tissue-Tek 4583; Sakura Finetek USA, Torrance, CA, USA), frozen, and cut into 10 μm sections at −23 °C. After all the samples were completely sectioned, ten slides were selected out of the beginning, middle, and end part of each sample. Sections were stained with hematoxylin and eosin (H and E) and Masson’s trichrome (MT) to examine muscle degeneration and tissue inflammation.

Sections were subjected to immunofluorescent staining by anti-CD31 (BioLegend, San Diego, CA, USA) and anti-smooth muscle (SM) α-actin. Fluorescein isothiocyanate-conjugated secondary antibodies (Jackson ImmunoResearch Laboratories, West Grove, PA, USA) were used to visualize the signals. The sections were counterstained with DAPI and examined under a fluorescence microscope (Nikon TE2000, Tokyo, Japan). Five pictures were randomly selected from each slide and fluorescent vessels were counted to quantify CD31 and SM α-actin positive vessels in ischemic regions. Fluorescent vessels were calculated as vessel number per mm^2^.

### 2.13. Statistical Analysis

GraphPad Prism 7 software was used for all statistical analysis in this study. Triplicate data were analyzed using one-way analysis of variance (ANOVA) with a Bonferroni test in all the experiments. Comparisons between two independent samples were performed using a two-tailed Student’s test. A *p* value of <0.05 was considered statistically significant. Results are expressed as the mean ± standard deviation for all quantitative analyses.

## 3. Results

### 3.1. Polymer Synthesis and Characterization

**Figure 2:**
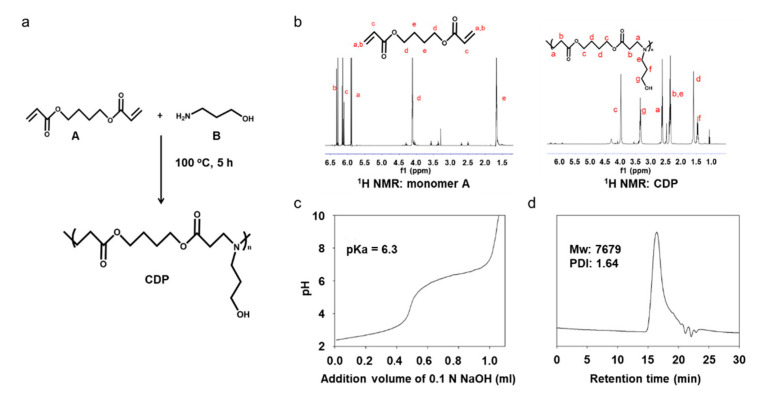
Synthesis and characterization of CDP. (**a**) Synthesis scheme for CDP production. The polymer composition was confirmed by (**b**) ^1^H NMR spectra for monomer A and CDP. (**c**) Titration curve and (**d**) GPC of polymer CDP.

The procedure for CDP synthesizing is illustrated in Figure 2a.

CDP was synthesized by Michael-addition polymerization of monomer A and B. The activated double bond in monomer A was the combined amine in compound B by Michael-type step polymerization. The structure of the polymer was checked by ^1^H NMR, as shown in Figure 2b. Proton signals of the double bond (peaks a, b, and c in Figure 2b monomer A) disappeared in CDP spectra (Figure 2b CDP), which demonstrated that the monomer A substrate was totally consumed by this synthesis. In Figure 2b CDP, proton signals at 2.65 (peak a), 2.35 (peak b), 4.0 (peak c), and 1.65 ppm (peak d) were assigned to monomer A, and signals at 2.35 (peak e), 1.5 (peak f), and 3.4 ppm (peak g) were assigned to monomer B. The signals for monomer A and B shown in the spectrum for CDP indicate that polymerization occurred using monomer A and B without the use of other chemicals. From the NMR analysis of the monomer-mixed CDP produced after the synthesis of monomer A and B, we concluded that the conversion ratio reached 97.8%. The pKa, the point at which CDP switched from hydrophobic to hydrophilic, was confirmed to be 6.3 (Figure 2c), and the molecular weight of CDP was 7679 as measured by GPC (Figure 2d).

### 3.2. Analogous Cell Adhesion, Growth Rate, and Viability between hADSCs Cultured on NCDs and CDP-Coated Culture Dishes

**Figure 3:**
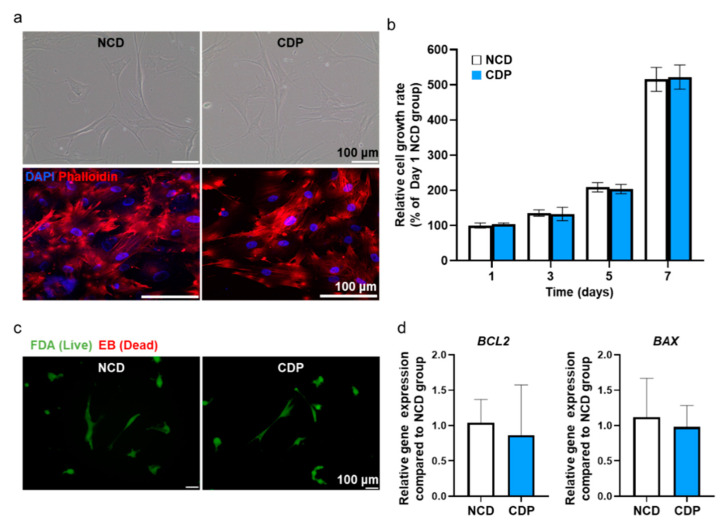
Adhesion, viability, and apoptotic activity in the hADSCs cultured on the CDP-coated culture dish. (**a**) Representative light microscopy images of hADSCs cultured on the NCD and CDP (scale bar = 100 μm). Fluorescence images of phalloidin (red) in the NCD and CDP-cultured hADSCs. The nuclei were stained with DAPI (blue). Scale bar = 100 μm. (**b**) Cell growth rate of the hADSCs cultured on the NCDs and CDP evaluated by the CCK-8 assay. (**c**) Fluorescence images of NCD and CDP-cultured hADSCs stained with FDA and EB on day 1. Green and orange-red colors indicate viable and dead cells, respectively, scale bar = 100 μm. (**d**) Anti-apoptotic (*BCL2*) and pro-apoptotic (*BAX*) gene expression of NCD and CDP-cultured hADSCs on day 1.

To evaluate whether the CDP coating affects the cell adhesion, hADSCs were seeded and cultured on a dish coated with CDP, and the results were then compared with the normal cell culture dishes (NCDs). Both the optical and fluorescent images showed that the cells cultured on the CDP-coated culture dishes showed no abnormality in terms of cell adhesion compared with the cell cultured on the NCDs (Figure 3a).

Phalloidin staining showing F-actin in the cells showed that the cytoskeleton in the hADSCs cultured on the CDP-coated culture dishes was similar to that in the cells cultured on the NCDs. Effects of CDP coating on the cell growth rate in hADSCs were also examined using the CCK-8 assay, and the data are expressed as a percentage relative ratio to the cells in NCDs at day 1 (Figure 3b). At 1, 3, 5, and 7 days after culturing the hADSCs on the CDP-coated culture dishes, the cell growth rate showed similar results when compared with the NCDs group. In addition, cell viability was confirmed using FDA/EB staining and qRT-PCR assay. The FDA/EB staining assay showed that there was no difference in cell viability in both groups on day 1 (Figure 3c). The expression of *BCL-2* (anti-apoptotic) and *BAX* (pro-apoptotic) quantified by qRT-PCR also showed no significant difference between the two groups (Figure 3d).

### 3.3. Similar Cellular Function between NCPs and CDP-Coated Culture Dishes after Cell Detachment

**Figure 4:**
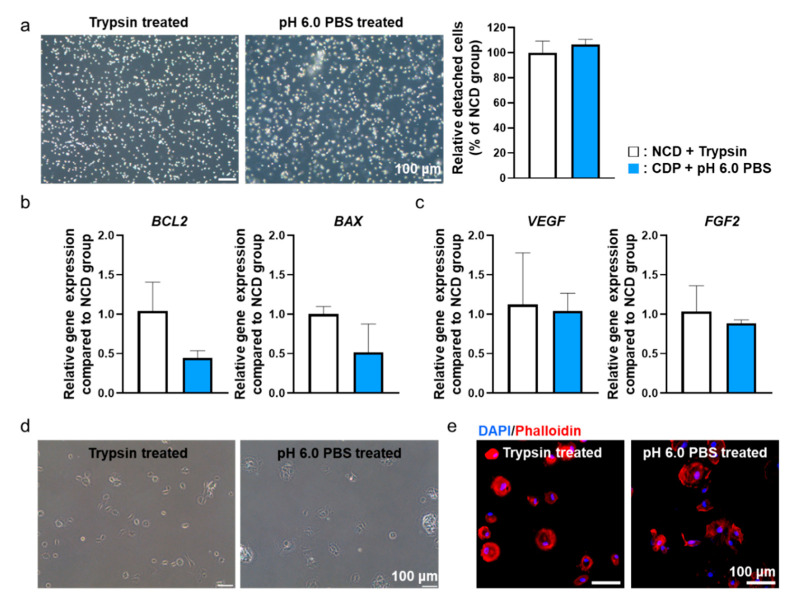
pH 6.0 PBS-treated hADSCs cultured on the CDP-coated culture dishes showed similar behavior compared to trypsin-treated hADSCs cultured on the NCD. (**a**) Light microscope images of trypsin-treated hADSCs cultured on the NCD (NCD + Trypsin) and pH 6.0 PBS-treated hADSCs cultured on the CDP-coated culture dishes (CDP + pH 6.0 PBS). Scale bar = 100 μm. (**b**) Anti-apoptotic (*BCL2*) and pro-apoptotic (*BAX*) gene expression of NCD + Trypsin and CDP + pH 6.0 PBS hADSCs. (**c**) Angiogenic paracrine factors (*VEGF* and *FGF2*) expression of NCD + Trypsin and CDP + pH 6.0 PBS hADSCs. (**d**) Light microscope re-adhesion images of NCD + Trypsin and CDP + pH 6.0 PBS hADSCs after re-attachment. (**e**) Fluorescence images of phalloidin (red) in NCD + Trypsin and CDP + pH 6.0 PBS hADSCs after re-attachment. The nuclei were stained with DAPI (blue). Scale bar = 100 μm.

In order to confirm whether pH 6.0 PBS could detach the hADSCs cultured on the CDP-coated culture dishes, we compared the cells-treated CDP-coated dishes with conventional trypsin-EDTA treatment. (Trypsin-EDTA treated hADSCs cultured on the normal cell culture dishes (NCD + Trypsin) and pH 6.0 PBS treated the hADSCs cultured on the CDP-coated culture dishes (PAE + pH 6.0 PBS).) Before detaching the hADSCs from the CDP-coated culture dishes, we removed the culture medium and washed the cells with pH 7.4 PBS. Then, to induce the detachment of the hADSCs from the CDP-coated culture dishes, we treated the cells with pH 6.0 PBS for 10 min at 37 °C. In optical images, we confirmed that the cells in both groups had a round shape morphology. The number of detached hADSCs was quantified by cell counting and there was no difference in both groups (Figure 4a).

In addition, to verify the effect of pH 6.0 PBS treatment on cellular function, we detached cells from CDP-coated culture dishes using pH 6.0 PBS and then allowed them to re-attach. The cellular functions were compared with the cells re-attached after detachment from cell culture dishes using trypsin-EDTA. Two hours after re-attachment, the cells from both groups were analyzed to investigate any changes in cellular function. Gene expression from the re-attached hADSCs related to apoptosis factors (BCL-2, BAX) (Figure 4b) and angiogenic paracrine factors (VEGF, FGF2) (Figure 4c) were not significantly different between the two groups. In addition, the form of re-attached cells in both groups were analyzed through optical images (Figure 4d) and phalloidin staining (Figure 4e). As shown in Figure 4d,e, no significant differences were observed between the two groups in terms of cell morphology. Similar results were found from CDP-coated culture dishes with human dermal fibroblast and HeLa cells (Figure S1).

### 3.4. Ischemic Hindlimb Recovery after Injecting hADSCs Harvested from NCDs and CDP-Coated Culture Dishes

**Figure 5:**
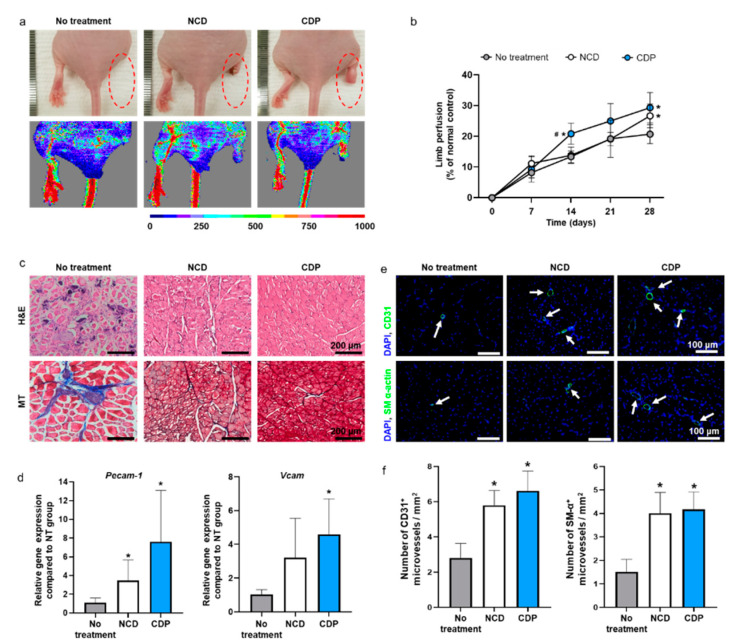
In vivo hindlimb ischemic disease treatment with the hADSCs collected from the NCD + Trypsin and CDP + pH 6.0 PBS. (**a**) Representative photographs and laser Doppler imaging of limbs taken on day 28 after hADSCs injection. (**b**) Limb perfusion on days 0, 7, 14, 21, and 28 day after hADSCs injection (^#^ *p* < 0.05 compared to NCD group and * *p* < 0.05 compared to no-treatment group). (**c**) Representative images of hematoxylin and eosin (H and E) and Masson’s trichrome (MT) staining in the hindlimb regions on day 28. Scale bar = 200 μm. (**d**) Relative expression of *Pecam-1* and *Vcam* in the hindlimb regions on day 28 (* *p* < 0.05 compared to no-treatment group). (**e**) Representative images of CD31 or SM-α-actin expression (green fluorescence) and DAPI (blue) staining in the hindlimb regions on day 28 (scale bar = 100 μm: White arrows indicate microvessels). (**f**) Number of CD31^+^ and SM-α^+^ microvessels in the hindlimb regions on day 28 (* *p* < 0.05 compared to no-treatment group).

Ischemic hindlimb recovery facilitated by the hADSCs cultured on the CDP-coated culture dishes and dissociated with pH 6.0 PBS was evaluated using a mouse ischemic hindlimb model. We transplanted hADSCs dissociated from the cell culture dishes with trypsin-EDTA or the CDP-coated culture dishes with pH 6.0 PBS into the gracilis muscle. On day 28, the hADSCs detached from the cell culture dishes using trypsin-EDTA (NCD group) and the CDP-coated culture dishes with pH 6.0 PBS (CDP group) showed significantly improved ischemic limb salvage and limb perfusion than the no-treatment group (Figure 5a,b).

Limb perfusion was measured on days 0, 7, 14, 21, and 28 after treatment through laser Doppler imaging analysis. Limb perfusion is represented as a percentage of the normal control (Figure 5b). However, there was no significant difference in blood flow on day 28 between the NCD group and CDP group (Figure 5b). In addition, the NCD and CDP groups showed improvements in ischemic tissue regeneration compared to the no-treatment as evidenced by H and E and (Masson’s trichrome) MT staining results (Figure 5c). The expression of *Pecam-1* and *Vcam*, both related to the presence of microvessels, on the ischemic limb was quantified by qRT-PCR. mRNA expression of *Pecam-1* in both the NCD and CDP groups were significantly upregulated in comparison with the no-treatment. mRNA expression of *Vcam-1* in the CDP groups showed a significant increment compared with the no-treatment. However, no significant differences were found between the NCD and CDP groups in any of parameters tested. The number of microvessels in the ischemic tissues was analyzed by immunohistochemistry (IHC) (Figure 5e) and quantified (Figure 5f). The number of CD31^+^ and SM-α actin^+^ microvessels were abundant in both the NCD and CDP groups compared to those in the no-treatment group, whereas no significant difference was found between NCD and CDP groups (Figure 5e,f).

## 4. Discussion

In this study, we showed an alternative method for harvesting stem cells through enzyme-free desorption using a pH-sensitive CDP instead of the conventional trypsin-EDTA method. Trypsin-EDTA is one of the representative enzymes that can decompose cell adhesion molecules for cell detachment from the cell culture dish [19]. Treating trypsin-EDTA for cell detachment requires fine control as its activity is highly affected by temperature and concentration of trypsin-EDTA. Additionally, excessive amounts of trypsin-EDTA treatment to cells may cause cellular damage, whereas insufficient trypsin treatment may decrease the yield of cell collection [20,21]. Moreover, according to the recent studies, enzymatic cell detachment can reduce the expression of cell surface antigen temporarily or stimulate unwanted differentiation of stem cells after successive cell cultures [22,23,24]. In particular, trypsin reduced the integrin-mediated cell adhesion of human adult stem cells and damaged the cell transmembrane receptor proteins of human adult stem cells. In addition, repetitive subculturing using trypsin reduced the proliferation rate, stemness-related gene expression, and differentiation ability of the stem cells [24]. Thus, we applied CDP as an alternative material for enzyme-free cell detachment that can show similar cell detachment compared to trypsin-EDTA treatment but without potential cellular damages.

We have cultured and detached the hADSCs from CDP-coated cell culture dishes and compared the cellular effects with conventional trypsin-EDTA treatment. The CDP synthesized to coat the surface of the cell culture dish was developed from PAE and showed pH-sensitive degradation under pH 6.0 PBS. CDP was successfully synthesized with the polymerization occurring between the amine group and double bond of monomers A and B, as shown in Figure 2a. The pKa value of CDP was about 6.3 (Figure 2c), and thus, we expected the CDP to be dissolved to pH 6.0 aqueous solution. The hADSCs cultured on the surface of CDP-coated cell culture dishes showed similar cell adhesion and cell viability compared to the hADSCs cultured on NCDs (Figure 3). The hADSCs detached with pH 6.0 PBS from CDP-coated cell culture dishes successfully and in vitro cellular behaviors of re-attached hADSCs were evaluated again to confirm the maintenance effect of our CDP-based cell detachment system on the therapeutic efficacy. As shown in Figure 4, there was no difference in cell viability, angiogenic paracrine factor secretion, and adhesion morphology between hADSCs detached with trypsin-EDTA and the CDP system. In accordance with the in vitro results, the in vivo experiments using the hindlimb ischemia model evaluating angiogenic efficacy of the hADSCs detached from CDP-coated cell culture dishes showed similar limb salvage (Figure 5a,b), histological improvement (Figure 5c), and angiogenesis (Figure 5d–f) compared to the hADSCs detached with trypsin-EDTA. Collectively, applying a CDP coating to the cell culture dish for the hADSC culture and pH 6.0 PBS treatment for hADSC collection via CDP degradation can be considered a suitable alternative method for trypsin-EDTA treatment. Additionally, it has been reported that three-dimensionally cultured cells show more similar cellular behaviors found under the in vivo condition compared to those of two-dimensionally cultured cells. Three-dimensional cell culture platforms such as a three-dimensional hydrogel matrix might provide more similar physiological in vivo conditions as confirmed by cellular morphology and gene expressions [25,26,27]. The CDP of this study was applied to the cell detachment system simply through surface-coating. Therefore, combining the CDP system with three-dimensional cell culture systems might suggest an advanced cell culture platform in future research.

Simple and easy cell detachment using the CDP in this study is less dependent on specific conditions such as temperature. Moreover, cellular behaviors of hADSCs cultured on CDP and detached with pH 6.0 PBS showed no differences against the cells cultured on the normal cell culture dish and detached with trypsin-EDTA. Additionally, it is relatively convenient to coat CDP on the surface of the cell culture dish compared to other polymers developed for cell detachments [8,9,10]. Compared to the previously reported nanoparticles or polymer used in cell detachment systems, CDP requires a relatively simple manufacturing process and low cost. In addition, the cell detaching method developed in this study is less likely to damage cells by pH change as the CDP can be removed through washing with mild pH conditions compared to the preexisting methods. Furthermore, our CDP method is free from potential problems such as genetic mutations and low cell viability that can be caused by nondegradable nanoparticles or substrates. CDP can also be applied to both single-cell detachment and special types of cell detachment such as cell sheets easily. Taken together, our CDP may suggest a new platform for enzyme-free cell detachment for future cell culture systems and tissue engineering.

## 5. Conclusions

In summary, we showed that we were able to retrieve the hADSCs from cell culture dishes in an enzyme-free manner through a mild pH condition by applying CDP to the cell culture system. The hADSCs cultured on CDP-coated culture dishes and detached through pH 6.0 PBS had similar cellular functions to the cells cultured on cell culture dishes and detached with trypsin-EDTA. Additionally, we also confirmed the similar in vivo therapeutic angiogenesis from both hADSCs retrieved from CDP-coated culture dishes, and normal cell culture dishes with enzyme treatment. Our CDP system may provide a new alternative stem cell harvesting platform to cell therapies.

## Figures and Tables

**Table 1:** Primers used for quantitative real-time (qRT)-PCR analyses.

Gene	Primer
Human *GAPDH*	sense 5′-GTC GGA GTC AAC GGA TTT GG-3′
	antisense 5′-GGG TGG AAT CAA TTG GAA CAT-3′
Human *BCL2*	sense 5′-CTT GAC AGA GGA TCA TGC TGT AC-3′
	antisense 5′-GGA TGC TTT ATT TCA TGA GGC-3′
Human *BAX*	sense 5′-CAT GTT TTC TGA CGG CAA CTT C-3′
	antisense 5′-AGG GCC TTG AGC ACC AGT TT-3′
Human *VEGF*	sense 5′-GAG GGC AGA ATC ATC ACG AAG T-3′
	antisense 5′-CAC CAG GGT CTC GAT TGG AT-3′
Human *FGF2*	sense 5′-AGC GGC TGT ACT GCA AAA AC-3′
	antisense 5′-GTA GCT TGA TGT GAG GGT CG-3′
Mouse *β-actin*	sense 5′-GGC TGT ATT CCC CTC CAT CG-3′
	antisense 5′-CCA GTT GGT AAC AAT GCC ATG T-3′
Mouse *Pecam-1*	sense 5′-CAA ACA GAA ACC CGT GGA GAT G-3′
	antisense 5′-ACC GTA ATG GCT GTT GGC TTC-3′
Mouse *Vcam-1*	sense 5′-CTG GGA AGC TGG AAC GAA GT-3′
	antisense 5′-GCC AAA CAC TTG ACC GTG AC-3′

## Data Availability

Data available on request due to restrictions, e.g., privacy or ethical.

## Supplementary Materials

The following are available online at https://www.mdpi.com/1996-1944/14/3/491/s1, Figure S1. hDF and HeLa treated with PBS at pH 6.0 cultured on the CDP-coated culture dishes showed similar behavior compared to trypsin treated hDF and HeLa cultured on NCDs. (a) Representative light microscope images of hDF and HeLa cultured on the NCD and CDP. Scale bar = 500 μm. (b) Representative light microscope images of trypsin treated hDF and HeLa cultured on the NCD (Trypsin treated) and pH 6.0 PBS treated hDF and HeLa cultured on the CDP-coated culture dishes (pH 6.0 PBS treated). Scale bar = 500 μm. (c) Fluorescence images of phalloidin (red) in trypsin treated and pH 6.0 PBS treated hDF and HeLa after re-attachment. The nuclei were stained with DAPI (blue). Scale bar = 100 μm. (d) Cell viability rate of hDF and HeLa cultured on the NCDs and CDP evaluated by the CCK-8 assay. (e) Relative amount of detached hDF and HeLa was quantified by cell counting.
