# Supplementary material for: Development of pH-Responsive Polymer Coating as an Alternative to Enzyme-Based Stem Cell Dissociation for Cell Therapy
Source: Materials (Basel). 2021 Jan 20;14(3):491. doi: 10.3390/ma14030491 (PMC7864336; doi:10.3390/ma14030491)
Supplement: Supplementary file 1 [file materials-14-00491-s001.zip › materials-1072972-supplementary.pdf]

materials

Supplementary Information

Development of pH-responsive polymer coating as an alternative to enzyme-based stem cell dissociation for cell therapy

Materials and Methods

Cell culture

The human dermal fibroblast (hDF) and HeLa were purchased from Lonza (Walkersville, MD, USA) and cultured in Dulbecco's Modified Eagle's Medium (DMEM, Gibco BRL, Gaithersburg, MD, USA) supplemented with 1 % (v/v) penicillin (Gibco BRL, Waltham, MA, USA), and 10 % (v/v) fetal bovine serum (Gibco BRL, Waltham, MA, USA). All experiments were performed on hDF with less than six passages.

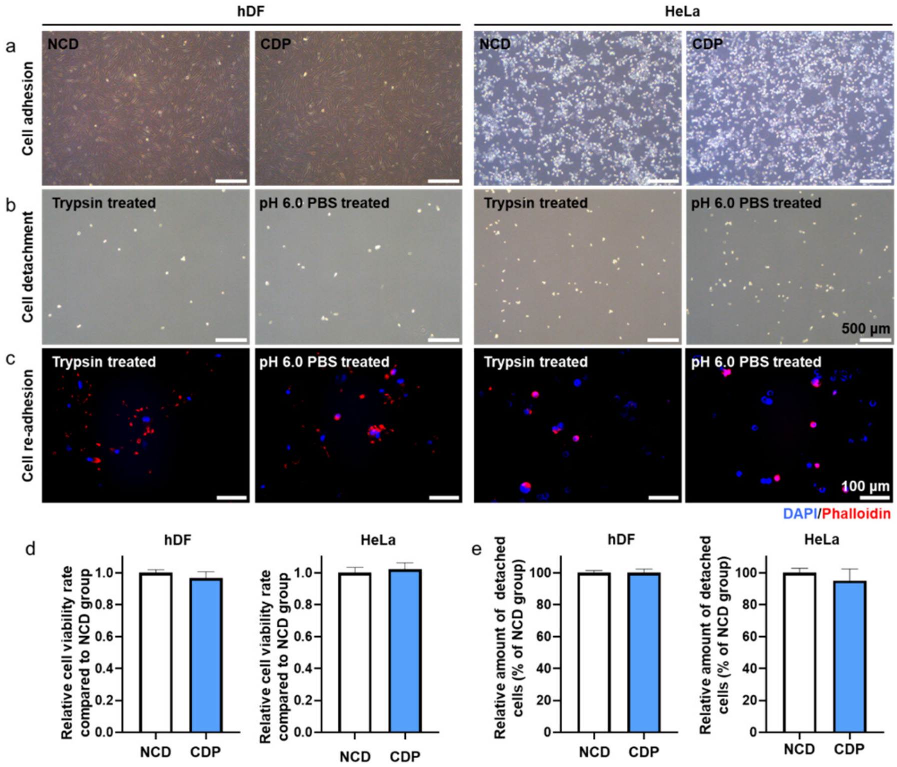

Scheme 1. hDF and HeLa treated with PBS at pH 6.0 cultured on the CDP-coated culture dishes showed similar behavior compared to trypsin treated hDF and HeLa cultured on NCDs. (**a**) Representative light microscope images of hDF and HeLa cultured on the NCD and CDP. Scale bar = 500 µm. (**b**) Representative light microscope images of trypsin treated hDF and HeLa cultured on the NCD (Trypsin treated) and pH 6.0 PBS treated hDF and HeLa cultured on the CDP-coated culture dishes (pH 6.0 PBS treated). Scale bar = 500 µm. (**c**) Fluorescence images of phalloidin (red) in trypsin treated and pH 6.0 PBS treated hDF and HeLa after re-attachment. The nuclei were stained with DAPI (blue). Scale bar = 100 µm. (**d**) Cell viability rate of hDF and HeLa cultured on the NCDs and CDP evaluated by the CCK-8 assay. (**e**) Relative amount of detached hDF and HeLa was quantified by cell counting.
